# Fetal brain five-axial planes and 3D-ICRV cerebral surface imaging from 16 to 36 weeks of gestation

**DOI:** 10.1186/s12880-026-02643-2

**Published:** 2026-08-11

**Authors:** Huaxuan Wen, Yimei Liao, Bing Wang, Dandan Luo, Xiaohong Zhong, Christoph C. Lees, Shengli Li

**Affiliations:** 1 Shenzhen Maternity and Child Healthcare Hospital, Women and Children’s Medical Center, Southern Medical University, Shenzhen, Guangdong China; 2 Peking University Shenzhen Hospital, Shenzhen, Guangdong China; 3 Women and Children’s Hospital, School of Medicine, Xiamen University, Xiamen, Fujian Province 361000 China; 4 Imperial College London, London, UK

**Keywords:** Fetal, Brain, 3D-ICRV, Cerebral surface, Neurosonography, Cortical development

## Abstract

**Background:**

Recent advances in fetal neurosonography have increased interest in prenatal cortical assessment. However, the transvaginal approach, while providing superior resolution, is technically complex and limited to specialized settings.

**Methods:**

This study utilizes a transabdominal approach combining a five-axial-plane protocol and 3D Inversion Crystal Realistic Vue (3D-ICRV) rendering technique to evaluate fetal cortical development.

**Results:**

We present high-definition ultrasound images and schematic diagrams, selected from 157 prospectively acquired volumes of 21 normal fetuses (16–36 weeks), illustrating the developmental progression of the Sylvian fissure and other primary sulci.

**Conclusion:**

This pictorial guide may serve as a practical, accessible reference for sonographers and fetal medicine specialists in the assessment of fetal cerebral cortex maturation.

**Supplementary Information:**

The online version contains supplementary material available at https://doi.org/10.1186/s12880-026-02643-2.

## Introduction

Recent advances in fetal neurosonography, particularly in visualizing the cerebral cortex, have heightened clinical interest in prenatal cortical assessment. While transvaginal neurosonography provides superior resolution, its technical complexity limits its routine application to high-risk pregnancies, typically performed by specialists in dedicated neurosonography clinics [1]. Consequently, the initial cortical evaluation in most low-risk pregnancies continues to rely on the transabdominal approach by sonographers. In this study, we integrate a five-axial-plane protocol with 3D-ICRV rendering within the transabdominal approach, and characterize the corresponding imaging features of fetal cortical development.

## Methods

### Five-axial-plane assessment

To facilitate a more comprehensive fetal brain assessment via this accessible transabdominal method, we previously proposed extending the standard three-axial-plane protocol (encompassing the transventricular, transthalamic, and transcerebellar planes) to include two additional planes: the transcalvarial and the trans-genu-and-splenium planes [2]. This five-axial-plane protocol also permits the preliminary evaluation of major sulci: the parieto-occipital sulcus (POS), cingulate sulcus (CgS), central sulcus (CS), pre-/postcentral sulcus, intraparietal sulcus (IPS), and superior frontal sulcus (SFS) on the transcalvarial and trans-genu-and-splenium planes; and the Sylvian fissure (SF) along with the inferior frontal sulcus (IFS) and superior/inferior temporal sulcus (STS/ITS) on the transventricular, transthalamic, and transcerebellar planes.

### 3D-ICRV surface imaging

In addition, we applied the 3D Inversion Crystal Realistic Vue (3D-ICRV) rendering technology [3], which enables intuitive visualization of the cerebral sulci on the convex surface of the fetal cerebral hemispheres. This technique provides a stereoscopic view that closely resembles a gross anatomical perspective, allowing detailed assessment of sulcal morphology and surface topography.

### Ultrasound acquisition and post-processing

Neurosonography used a W10 system (Samsung Medison, Seoul, Korea) with a 1–8 MHz curvilinear transducer, and post-processing was performed using the system’s built-in software. The initial acquisition plane is the transventricular or transthalamic plane, with the ultrasound beam ideally directed through the coronal, squamous sutures and the sphenoid fontanelle to reduce calvarial shadowing [4]. Volumes showing optimal delineation of cortical sulci and gyri are selected for post-processing using Crystal Vue, Realistic Vue, and Inversion mode—functions fully automated on the Samsung W10 system and also available on other platforms with 3D surface rendering and inversion capabilities. To optimize 3D-ICRV display, follow these steps. Select a scanning depth based on gestational age and moderately increase the 2D gain. Keep the probe steady during the automatic sweep. Position the ROI cropping line between the brain surface and the calvarial rim, ensuring the rendering window captures the cortical surface. Finally, adjust the light source to cast shadows that enhance fine sulci, yielding a more stereoscopic surface image.

High-quality 3D-ICRV imaging requires clear 2D fetal brain images and is feasible with any fetal lie (cephalic, breech, or transverse)—unlike transvaginal neurosonography, which depends on cephalic presentation [5]. The overall success rate is ~ 91% [3]. Limiting factors include fetal motion, maternal breathing, abdominal wall thickness, late-gestation cranial ossification, and maternal pelvic shadowing from a low-lying fetal head.

## Results

Twenty-one healthy pregnant women were included in this study. Mean maternal age and BMI were 33.29 ± 4.16 years and 26.19 ± 3.03, respectively. All mothers were healthy, with no pregnancy-related disorders (e.g., diabetes, hypertension), and routine prenatal examinations were normal. Fetal growth parameters were consistent with gestational age confirmed by menstrual dating or first-trimester crown-rump length correction. Deliveries were by elective cesarean section or vaginal delivery, with mean gestational age at delivery of 39.05 ± 0.95 weeks and mean birth weight of 3307.14 ± 361.58 g. No delivery complications, abnormal neonatal physical examinations, or adverse follow-up (to 2 years of age) were noted.

**Fig. 1:**
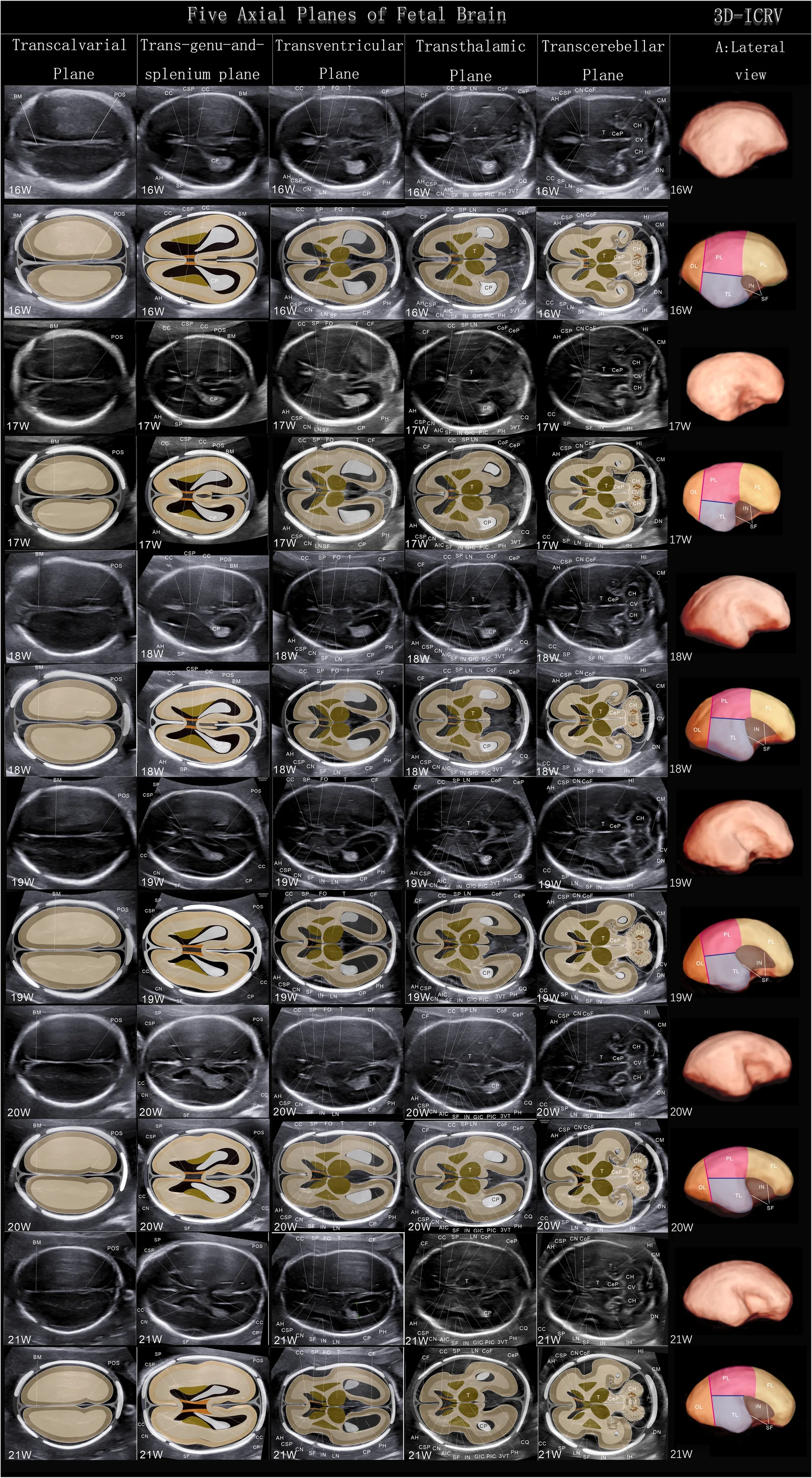

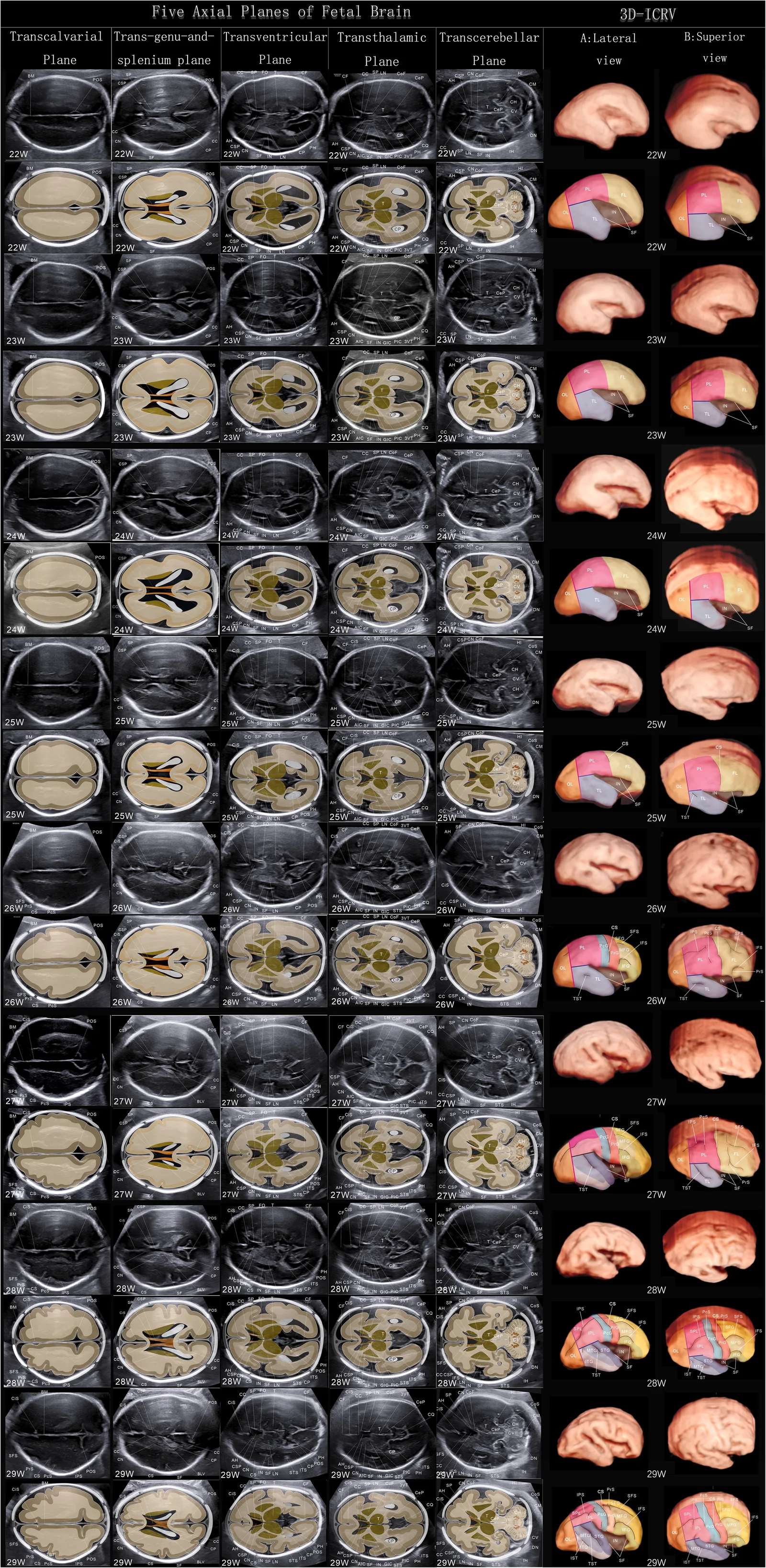

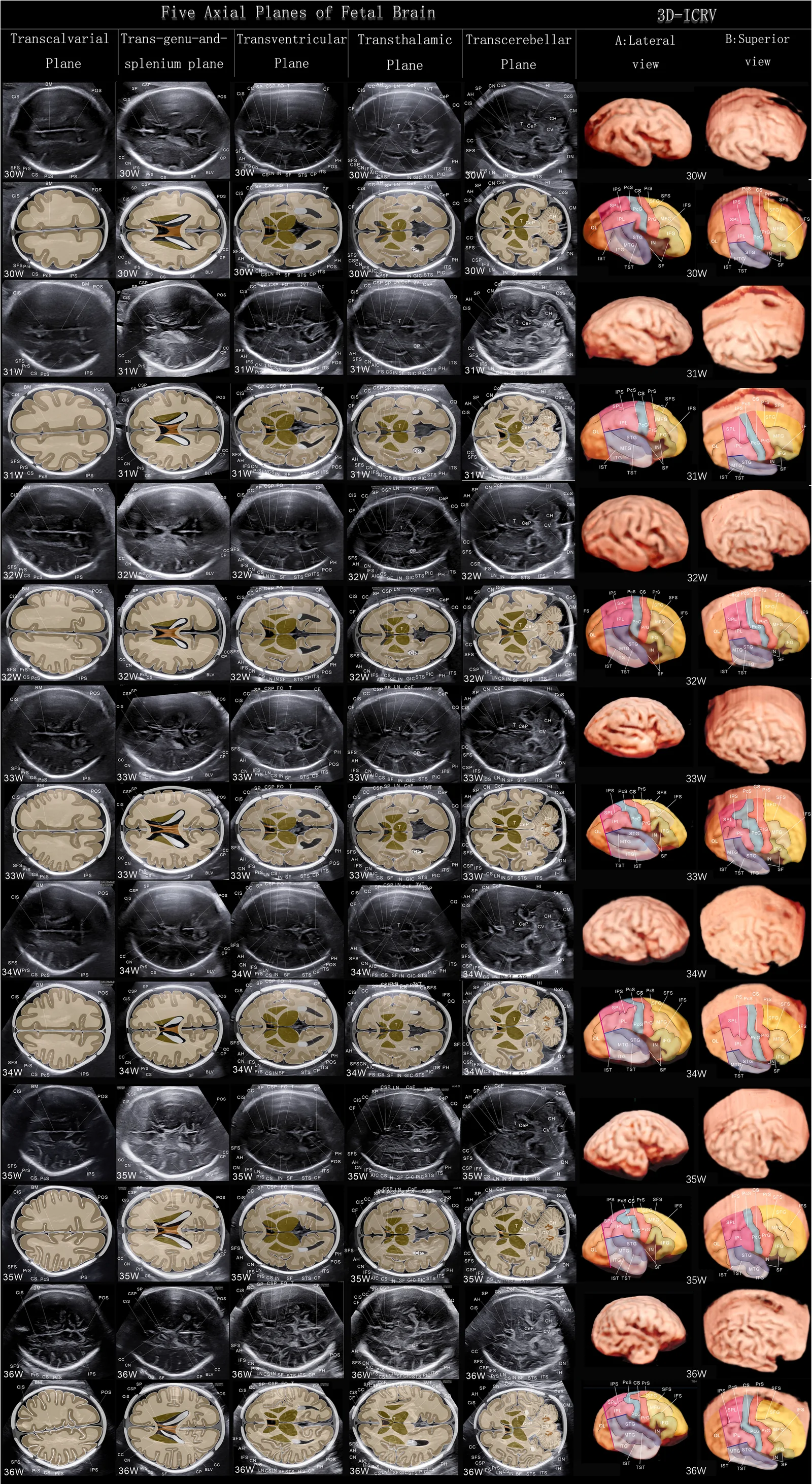
Five axial planes and 3D-ICRV of the normal fetal brain at 16-21weeks (**A**), 22-29weeks (**B**), 30-36weeks (**C**). Sequential ultrasound images and schematic diagrams demonstrating the developmental progression of cerebral sulcation and gyration in the fetus (detailed descriptions are provided in the main text)

From a total of 157 prospectively acquired cerebral ultrasound volumes, we selected the most representative, high-quality images from 21 normal fetuses. For each fetus, multiple volumes were acquired, and the final images were chosen from the optimal acquisitions for that case. The developmental patterns observed in these fetuses were consistent with those in our previous 3D-ICRV study [3]. Finally, we present a series of high-resolution ultrasound images and schematic diagrams **(**Fig. 1**)**, including five axial planes and corresponding 3D-ICRV reconstructions of the fetal brain, obtained from normal fetuses at 16–36 weeks of gestation. The developmental patterns of sulci and gyri are detailed in the following sections.

**Table:** 

SF, Sylvian Fissure; CF, Cerebral Falx; CoS, Collateral Sulcus; CgS, Cingulate Sulcus; IN, Insula; CS, Central Sulcus; PrS, Precentral Sulcus; PcS, Postcentral Sulcus; PrG, Precentral Gyrus; PcG, Postcentral Gyrus; **FL**,** Frontal Lobe;** SFS, Superior Frontal Sulcus; IFS, Inferior Frontal Sulcus; SFG, Superior Frontal Gyrus; IFG, Inferior Frontal Gyrus; MFG, Middle Frontal Gyrus;	**PL**,** Parietal Lobe;** POS, Parieto-Occipital Sulcus; IPS, Intraparietal Sulcus; SPL, Superior Parietal Lobule; IPL, Inferior Parietal Lobule; **TL**,** Temporal Lobe;** STS, Superior Temporal Sulcus; ITS, Inferior Temporal Sulcus; STG, Superior Temporal Gyrus; ITG, Inferior Temporal Gyrus ; MTG, Middle Temporal Gyrus; **OL**,** Occipital Lobe** CH, Cerebellar Hemisphere; CM, Cisterna Magna; CV, Cerebellar Vermis;	CC, Corpus Callosum; GCC, Genu of Corpus Callosum; SCC, Splenium of Corpus Callosum; FO, Fornix; CoF, Column of the Fornix; SP, Septum Pellucidum; CSP, Cavum Septum Pellucidum CP, Choroid Plexus; PH, Posterior Horn of the Lateral Ventricle; AH, Anterior Horn of the Lateral Ventricle; IH, Inferior Horn of the Lateral Ventricle BLV, Body of the Lateral Ventricle;	T, Thalamus; CN, Caudate Nucleus; CQ, Cistern of Quadrigeminal; DN, Dentate Nucleus; CeP, Cerebral Peduncle; LN, Lentiform Nucleus; Hi, Hippocampus; BM, Midline of the Brain; AIC, Anterior limb of Internal Capsule; GIC, Genu of Internal Capsule; PIC, Posterior limb of Internal Capsule

### Developmental features of the Sylvian fissure

On 2D ultrasound, a simplified grading system for the SF on the transthalamic view was employed to delineate its developmental sequence from insular formation to operculization [6, 7]. These grading methods are highly useful for sonographers in understanding SF development. The SF typically evolves as follows: it appears as a curved depression after about 18–20 weeks; transitions to a plateau-like configuration around 23 weeks as the circular sulcus becomes more discernible; exhibits an acute angulation at the temporal operculum almost in all fetuses after 27 weeks; demonstrates opercular closure around 30 weeks; and presents identifiable insular gyri and sulci after 34 weeks. Based on our observations, the SF and parieto-occipital sulcus (POS) are detectable in most cases after 18 weeks.

On 3D-ICRV, the SF manifests as follows: Before 22–23 weeks, the Sylvian fissure appears as a shallow triangular indentation with a gradually enlarging area and a rounded apex. After 24 weeks, the triangular area progressively decreases and the apex becomes sharper. Following 27 weeks, the superior part of the triangle begins to close in a posterosuperior-to-anteroinferior direction. Between 30 and 35 weeks, the inferior frontal gyrus grows increasingly convoluted, leading to the gradual formation of an “M”-shaped structure in Broca’s area. Additionally, 3D-ICRV shows that the uncovered area of the insula gradually increases from 20 weeks, peaks at 25–26 weeks, and then decreases after 27 weeks as the opercula close and the insula becomes progressively covered.

### Developmental features of other primary sulci

On 2D ultrasound, other primary sulci develop rapidly between 26 and 30 weeks [8]: the central sulcus (CS) and superior temporal sulcus (STS) emerge at 25–26 weeks, followed by the pre- and postcentral sulcus, superior frontal sulcus (SFS), inferior frontal sulcus(IFS), and inferior temporal sulcus (ITS) after 26 weeks, with visualization rates increasing with gestational age. The cingulate sulcus (CgS), owing to its segmental development, may first appear on different axial planes. By 30 weeks, most primary sulci are observable on axial planes.

On 3D-ICRV, other sulci begin to emerge after 25 weeks. The first to appear are the central sulcus (CS) and the superior temporal sulcus (STS), around 25–26 weeks. The CS extends from superior to inferior and is consistently visible in 100% of cases by 27 weeks. The STS extends anteriorly from the parieto-occipital junction and is detectable in 95% of cases by 28 weeks. By 29 weeks, more than 90% of fetuses show the precentral sulcus (preCS), postcentral sulcus (postCS), superior frontal sulcus (SFS), inferior frontal sulcus (IFS), and inferior temporal sulcus (ITS). At 30 weeks, all primary sulci can be clearly identified. Beyond 30 weeks, sulcal shadows deepen and become more tortuous, with secondary sulci forming around them. By 35 weeks, the cortical surface resembles that of a term neonatal brain.

## Discussion

Current assessment of fetal cortical maturation relies on neurosonography and in utero MRI, yet no standardized method has been established [9–11]. Several expert-proposed approaches are used in select centers, including the Pistorius grading system [12] on transabdominal ultrasound or MRI, and measurement of the sylvian fissure angle on coronal planes obtained via transvaginal neurosonography [13]. These methods either analyze 2D planes directly or reconstruct specific planar images from 3D volumes that mimic conventional 2D slices. In contrast, 3D-ICRV technique provides a novel anatomical perspective—a direct, gross-anatomy-like view of the brain surface that is more intuitive and visually explicit.

According to ISUOG guidelines [1], fetal brain MRI is complementary to neurosonography. Yet, although MRI can generate surface reconstructions, its longer scan times, stricter motion requirements, and more demanding post-processing limit its practicality, leaving clinical practice largely 2D-based. 3D-ICRV offers a practical alternative: volume acquisition takes less than 5 s, post-processing within 5 min, and the procedure is reproducible, with suboptimal volumes (e.g., due to fetal movement or bony shadowing) immediately reacquirable.

These images may serve as a practical reference for sonographers and fetal medicine specialists in assessing the development of the fetal cerebral cortex. When abnormalities are suspected on these images, referral to a specialized fetal neurology clinic is recommended for further detailed evaluation. The integration of artificial intelligence may further enhance the precision of fetal cortical assessment [14, 15]. Accordingly, future prospective studies incorporating AI are needed to interpret 3D-ICRV volumes, enabling more precise and rapid evaluation of fetal cortical development. 

## Supplementary Information

Below is the link to the electronic supplementary material.

Supplementary Material 1

## Data Availability

The data and material in the present study are available from the corresponding author on reasonable request.

## Declarations

### Generative AI and AI-assisted technologies in the writing process

During the preparation of this work, the authors did not use generative AI and AI-assisted technologies in the writing process.
