# Supplementary material for: Fetal brain five-axial planes and 3D-ICRV cerebral surface imaging from 16 to 36 weeks of gestation
Source: BMC Med Imaging. 2026 Aug 11;26:599. doi: 10.1186/s12880-026-02643-2 (PMC13750044; doi:10.1186/s12880-026-02643-2)
Supplement: Supplementary file 1 [file 12880_2026_2643_MOESM1_ESM.pdf]

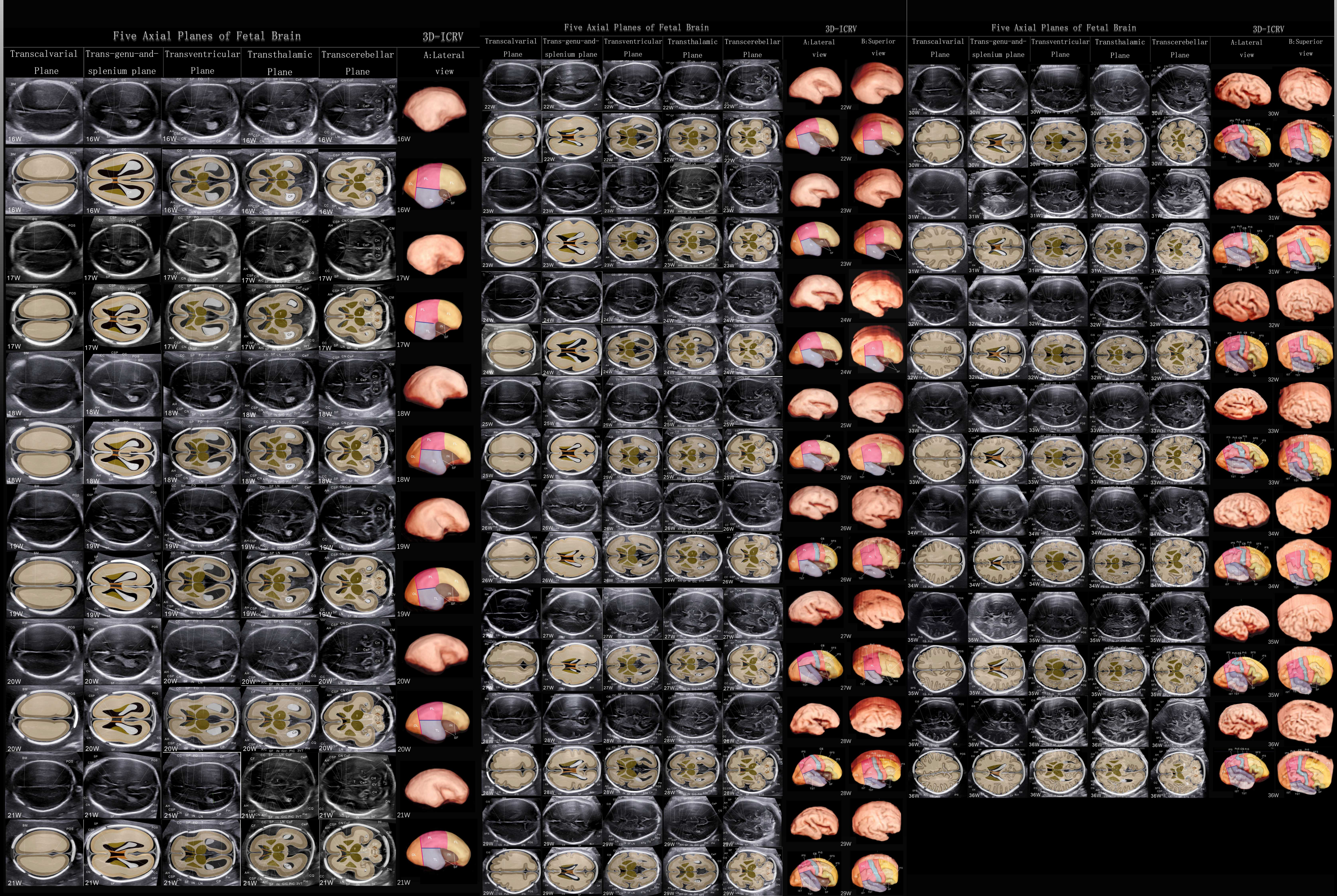
Five Axial Planes of Fetal Brain
3D-ICRV
Transcalvarial Plane
Trans-genu-and-splenium plane
Transventricular Plane
Transthalamic Plane
Transcerebellar Plane
A:Lateral view
B:Superior view
16W
17W
18W
19W
20W
21W
22W
23W
24W
25W
26W
27W
28W
29W
30W
31W
32W
33W
34W
35W
36W
